# The molecular landscape of *ASPM* mutations in primary microcephaly

**DOI:** 10.1136/jmg.2008.062380

**Published:** 2008-11-21

**Authors:** A K Nicholas, E A Swanson, J J Cox, G Karbani, S Malik, K Springell, D Hampshire, M Ahmed, J Bond, D Di Benedetto, M Fichera, C Romano, W B Dobyns, C G Woods

**Affiliations:** 1 Department of Medical Genetics, Cambridge Institute for Medical Research, University of Cambridge, Cambridge, UK; 2 University of Chicago, Department of Human Genetics, Chicago, Illinois, USA; 3 Department of Clinical Genetics, St James’s University Hospital, Leeds, UK; 4 Section of Ophthalmology and Neuroscience, Leeds Institute of Molecular Medicine, University of Leeds, Leeds, UK; 5 Laboratory of Genetic Diagnosis, I.R.C.C.S. Associazione Oasi Maria Santissima, Troina (EN), Italy; 6 Unit of Paediatrics and Medical Genetics, I.R.C.C.S. Associazione Oasi Maria Santissima, Troina (EN), Italy

## Abstract

**Background:**

Autosomal recessive primary microcephaly (MCPH) is a model disease to study human neurogenesis. In affected individuals the brain grows at a reduced rate during fetal life resulting in a small but structurally normal brain and mental retardation. The condition is genetically heterogeneous with mutations in *ASPM* being most commonly reported.

**Methods and results:**

We have examined this further by studying three cohorts of microcephalic children to extend both the phenotype and the mutation spectrum. Firstly, in 99 consecutively ascertained consanguineous families with a strict diagnosis of MCPH, 41 (41%) were homozygous at the MCPH5 locus and all but two families had mutations. Thus, 39% of consanguineous MCPH families had homozygous *ASPM* mutations. Secondly, in 27 non-consanguineous, predominantly Caucasian families with a strict diagnosis of MCPH, 11 (40%) had *ASPM* mutations. Thirdly, in 45 families with a less restricted phenotype including microcephaly and mental retardation, but regardless of other neurological features, only 3 (7%) had an *ASPM* mutation. This report contains 27 novel mutations and almost doubles the number of MCPH associated *ASPM* mutations known to 57. All but one of the mutations lead to the use of a premature termination codon, 23 were nonsense mutations, 28 deletions or insertions, 5 splicing, and 1 was a translocation. Seventeen of the 57 mutations were recurrent. There were no definitive missense mutations found nor was there any mutation/phenotype correlation. *ASPM* mutations were found in all ethnic groups studied.

**Conclusion:**

This study confirms that mutations in *ASPM* are the most common cause of MCPH, that *ASPM* mutations are restricted to individuals with an MCPH phenotype, and that *ASPM* testing in primary microcephaly is clinically useful.

Our most defining feature as a species is our brain with its large size and cognitive functions leading to our great adaptability.^1^ Many genes are involved in the growth of the developing human brain but the identification of those which have a non-redundant and crucial role has proved difficult. Autosomal recessive primary microcephaly (MCPH) has emerged as a model disorder in which to seek such genes, as it is a condition where fetal brain growth is significantly reduced (as is head size throughout life), brain architecture is normal, and there are no apparent abnormalities in other body systems.^2^^–^4 Therefore the genes that cause MCPH may be expected to have an important, noticeable and non-redundant role in neurogenesis, but not other developmental processes.^5^ MCPH is also a diagnosable cause of mental retardation, and one with a substantial recurrence risk of one in four in subsequent children.

The current diagnostic criteria for MCPH are: congenital microcephaly more than −3 SD below age and sex means; mental retardation but no other neurological finding, such as spasticity, seizures, or progressive cognitive decline; normal height and weight, appearance, and results on chromosome analysis and brain scan.^6^ Despite this, MCPH still remains a clinical diagnosis of exclusion. Further, the recurrence risk of the MCPH phenotype after one affected child (with careful elimination of differential diagnoses) in a non-consanguineous family is one in eight, and in a consanguineous family one in six.^7^^–^9 Our first aim in designing this study was to be able to diagnose MCPH with greater accuracy, particularly at an early age, and to examine if the current diagnostic criteria accurately reflect the phenotypic spectrum of the disorder.

MCPH can be caused by recessive mutations in up to seven genes.^4^ ^6^ Unexpectedly most of the known MCPH genes, *CDK5RAP2*, *ASPM* and *CENPJ* (better known as *CPAP*^10^), encode centrosomal proteins, highlighting the importance of the centrosome in neurogenesis.^3^ ^11^^–^13 Despite this, a common mechanism explaining the role of the MCPH genes in neurogenesis has yet to emerge. All four known MCPH proteins are also present in the midbody (the microtubular structure linking daughter cells at the final stage of cytokinesis) and have apparently diverse roles: microcephalin in DNA repair and chromosome condensation, CDK5RAP2 and CENPJ in centriole/centrosome replication, and ASPM in modulating the plane of cytokinesis in neural precursors.^14^^–^19 Mutations in the *ASPM* gene at the MCPH5 locus on chromosome 1q31.3 have been considered the most common cause of MCPH.^6^ ^20^ ^21^ Our second aim in this study was to determine the frequency of *ASPM* mutations in microcephalic individuals, delineate the spectrum of mutations seen in the *ASPM* gene, and evaluate the utility of sequencing this gene in clinical practice.

The primary feature of MCPH is microcephaly—a reduced occipitofrontal head circumference (OFC)—which is an imperfect indicator of microencephaly (a small brain). Ideally volumetric magnetic resonance imaging (MRI) studies should be performed to quantitate brain volume, but this is not available in current clinical practice, whereas head circumference charts are tried, tested and useful.^22^^–^24 The choice of a cut-off value for microcephaly is important but arbitrary. Half of mentally retarded individuals are microcephalic^7^ ^25^ and half of children with OFC of −2 SD or less have normal intelligence.^26^ To define microcephaly some authors use an OFC <2 SD below the mean for age and sex, which results in 2% of the general population being considered microcephalic.^27^ ^28^ Others use <3 SD so that 0.1% of the general population are microcephalic and most do have mental retardation. 7 24 29 Added to this are probable ethnic variations and differences in norms between growth charts. Because of all of these considerations we use an OFC of more than −3 SD to define microcephaly in this study, realising that it could exclude a small number of true microcephalics but by corollary be unlikely to include people with normal brain volumes or intelligence.

## METHODS

Three cohorts of microcephalic individuals were investigated. The first cohort (cohort 1) is of 99 consecutively ascertained consanguineous families of Pakistani or Arab origin seen by one author (CGW), the first 56 of which have been previously partly reported.^6^ Seventy-three families had multiple affected members and 26 consisted of a singleton child. No family was more distantly related than parents being second cousins. All fulfilled the current MCPH diagnostic criteria, with the exception that a brain MRI scan was only available in eight families (all showed no architectural anomalies). The second cohort (cohort 2) is of 27 non-consanguineous MCPH families of predominantly European origin, including five sib pairs. All had a normal brain scan and conformed to standard MCPH diagnostic criteria. The third cohort (cohort 3) is of 45 children (from 45 families) that was examined specifically to answer the questions, “Are the current MCPH clinical criteria too restrictive?” and its corollary, “Do *ASPM* mutations cause a wider phenotype than just MCPH?”. All 45 children had congenital microcephaly and mental retardation but there was no exclusion for the presence of epileptic fits, spasticity or other gross motor problems, growth problems, the presence of profound mental retardation or brain malformations. All had a brain scan examined by one author (WBD). The phenotypes of this cohort are given in supplemental table 1. DNA was obtained from all affected individuals and where possible their parents. Research ethics approval was given for these studies by the host institutions.

**Table 1:** All reported autosomal recessive primary microcephaly (MCPH) mutations in *ASPM*

Mutation	Predicted protein effect	Reported in more than one family?	Ethnic group	Reference
c.74delG	p.Arg25fs	No	Caucasian	This paper
c.297+1460_3391-242del21844	Loss of microtubular binding domain	No	Caucasian	This paper
c.349C>T	p.Arg117X	Yes	Caucasian, Indian	20, 37
c.440delA	p.Lys147fs	No	Caucasian	This paper
c.577C>T	p.Gln193X	No	Caucasian	This paper
c.719_720delCT	p.Ser240fs	Yes	Pakistani	3
c.1152_1153delAG	p.Ser384fs	No	Caucasian	This paper
c.1179delT	p.Pro393fs	No	Caucasian	This paper
c.1258_1264delTCTCAAG	p.Ser420fs	Yes	Pakistani	3*
c.1260_1266delTCAAGTC	p.Ser420fs	Yes	Pakistani	21*
c.1366G>T	p.Glu456X	No	Turkish	This paper
c.1406_1413delATCCTAAA	p.Asn469fs	No	Caucasian	This paper
c.1590delA	p.Lys530fs	No	Caucasian	This paper
c.1727_1728delAG	p.Lys576fs	No	Yemeni	37
c.1959_1961delCAAA	p.Asn653fs	Yes	Saudi Arabian, Caucasian	This paper, 37
c.1990C>T	p.Gln664X	No	Pakistani	37
c.2761-25A>G	Creates “AG” motif between branch site and splice acceptor site, exon 10 skipped, exon 11 frameshift with 30 novel aa then stop	No	Caucasian	This paper
c.2936+5G>T	Removes splice donor site, additional 2 aa then stop	No	Pakistani	37
c.2967G>A	p.Trp989X	No	Caucasian	This paper
c.3055C>T	p.Arg1019X	Yes	Caucasian	This paper
c.3082G>A	Removes splice donor site, additional 3 aa then stop	No	Pakistani	37
c.3188T>G	p.Leu1063X	No	Pakistani	This paper
c.3527C>G	p.Ser1176X	No	Jordanian	37
**c.3663delG**	p.Arg1221fs	Yes	Pakistani	37
c.3710C>G	p.Ser1237X	No	Caucasian	This paper
c.3741+1G>A	Removes splice donor site, additional 9 novel aa then stop	No	Caucasian	This paper
c.3796G>T	p.Glu1266X	No	African	This paper
c.3811C>T	p.Arg1271X	Yes	Dutch†, Asian	This paper, 37
c.3978G>A	p.Trp1326X	Yes	Indian, Pakistani	20, 21
c.4581delA	p.Gly1527fs	No	Pakistani	37
c.4795C>T	p.Arg1599X	No	Pakistani	37
c.4855_4856delTA	p.Tyr1619fs	No	Pakistani	This paper
c.5136C>A	p.Tyr1712X	No	Pakistani	37
c.5149delA	p.Ile1717fs	No	Pakistani	34
**c.6189T>G**	p.Tyr2063X	No	Yemeni	35
c.6335_6336delAT	p.His2112fs	No	Pakistani	This paper
c.7489_7493delTATAT	p.Tyr2497fs	No	Caucasian	This paper
c.7761T>G	p.Tyr2587X	Yes	Pakistani	This paper, 3
c.7782_7783delGA	p.Gln2594fs	Yes	Caucasian, Pakistani	This paper
**c.7859_7860delAG**	p.Gln2620fs	No	Arab	This paper
c.8130_8131delAA	p.Thr2710fs	No	Caucasian	This paper
c.8378delT	p.Met2793fs	Yes	Pakistani	This paper
c.8508_8509delGA	p.Gln2836fs	Yes	Pakistani	This paper, 34, 37
c.8844delC	p.Ala2948fs	No	Caucasian	This paper
c.9118_9119insCATT	p.Tyr3040fs	No	Pakistani	21
c.9159delA	p.Lys3053fs	Yes	Pakistani	3, 37
c.9178C>T	p.Gln3060X	Yes	Indian, Caucasian	This paper, 20
c.9190C>T	p.Arg3064X	Yes	Pakistani, Dutch	This paper, 37
c.9238A>T	p.Leu3080X	Yes	Pakistani	This paper, 21
c.9557C>G	p.Ser3186X	Yes	Pakistani	21, 37
c.9681delA	p.Thr3227fs	No	Pakistani	This paper
c.9730C>T	p.Arg3244X	No	Pakistani	34
c.9745_9746delCT	p.Leu3249fs	No	Pakistani	This paper
c.9754delA	p.Arg3252fs	No	Yemeni	37
c.9789T>A	p.Tyr3263X	No	Pakistani	This paper
c.9984+1G>T	Removes splice donor site, additional 29 novel aa then stop	No	Pakistani	37
c.10059C>A	p.Tyr3353X	No	Pakistani	34
Translocation	Loss of IQ and armadillo domains	No	European	33

aa, amino acids.

*These are the same 7 base pair deletion mutation. †c.3811C>T has been found in a total of 3 out of 5 Dutch families with MCPH.

Bold face indicates MCPH associated with epileptic fits.

The position of each mutation is given as the number of bases from the start codon of the reference sequence NM_018136.3.

We sought evidence of linkage to *ASPM* in all of the consanguineous families. Polymorphic microsatellite marker or SNP chip analysis of all affected members was performed using standard methods and the results were examined for homozygosity at the MCPH5 locus.^6^ We sought a homozygous locus size of >1 cM, based on our previous study of recessive consanguineous families with proven recessive gene mutations.^30^ Of the total 99 consanguineous families, in 41 all affected members were homozygous for the MCPH5 locus (33 multi-affected families and eight singletons). In the remaining consanguineous families, affected family members were heterozygous for the MCPH5 locus in the case of singletons, or discordant for a MCPH5 haplotype in the multi-affected families. These families were assumed unlinked and the possibility of compound heterozygosity ignored.^31^ No linkage analysis was performed in the second two cohorts. The *ASPM* gene was sequenced using genomic DNA of one affected individual from each of the 41 consanguineous families that were homozygous at the MCPH5 locus, and all of the cohort 2 and 3 families. All exons and splice sites were included. Our recommended primers are given in supplemental table 2. All mutations found were shown to segregate faithfully in the family, although the degree of intrafamilial microcephaly varied, as previously reported.^6^

## RESULTS

**Figure 1:**
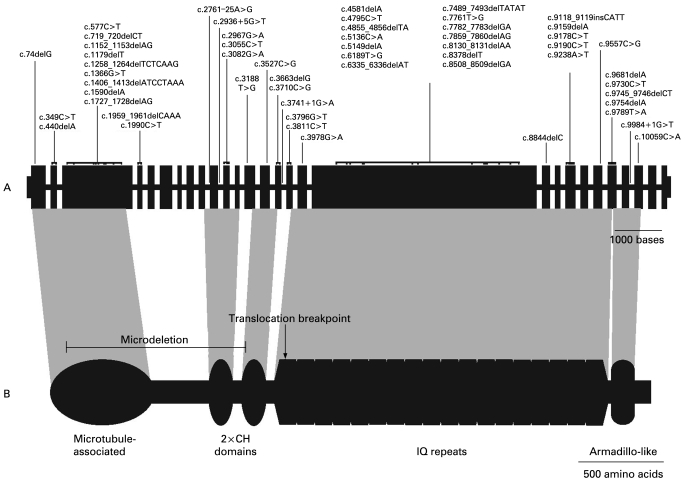
*ASPM* gene, protein and sites of autosomal recessive primary microcephaly (MCPH) mutations. (A) The exon/intron structure of the *ASPM* gene. Exons are scaled relative to each other, with exon 18 being the largest at 4.7 kb. Introns vary significantly in size, but for clarity are all shown as having the same arbitrary size. The position of the reported mutations is indicated, with italicised text denoting splicing mutations. All mutations are detailed in table 1. (B) The known and predicted domains of the ASPM protein: a microtubule binding domain; two calponin homology (CH) domains which are possibly responsible for transportation of the ASPM protein to the spindle poles; a region of 81 IQ/calmodulin binding domains; and a conserved armadillo-like C-terminal domain of unknown significance. The regions of the ASPM protein affected by the previously reported translocation breakpoint (Pichon *et al*^33^) and the microdeletion first reported here are shown by an arrow and a bar, respectively.

Of the consanguineous families in cohort 1, homozygous *ASPM* mutations were found in 39/41 of families (95% of families that were homozygous at the MCPH5 locus, 39% of all consanguineous families). Of the families that were homozygous at the MCPH5 locus with no *ASPM* mutations, one had a singleton affected child and one had an affected pair of first cousins. In cohort 2, of non-consanguineous, predominantly Caucasian individuals, *ASPM* mutations were found in 11/27 families (40%), and in 3/5 sib pairs. In this cohort 19 mutations were found: four were present in other cohorts, all but one led to a premature termination codon and no novel missense mutations were found. In three families the *ASPM* mutation was homozygous and the remainder were compound heterozygotes. In cohort 3, of 45 microcephalic individuals with a wider phenotype we found mutations in three families (7%). One consanguineous and one non-consanguineous singleton each had a homozygous nonsense mutation. Both of these homozygous mutations were present in other cohorts. A child from a third family carried two heterozygous protein truncating mutations. The mutations from all three cohorts and all previously reported mutations are described in table 1 and shown in fig 1.

## DISCUSSION

This work brings the total number of different *ASPM* mutations reported to 57. Of these mutations 17 are recurrent. All but one of the mutations reported here and previously is predicted to lead to the use of a premature termination codon in the *ASPM* gene (which has no common splice variants) (table 1). Of the 57 mutations 23 were nonsense mutations, 28 were small deletions or insertions leading to a change in the reading frame, and five were splice site mutations again leading to the use of a premature stop codon. In one family we found a large deletion of 21 844 bases with the loss of exons 2 through to 13, but which leaves the open reading frame intact. Cloning of the deletion showed a loss of part of intron 1 through to intron 13, and the breaks did not occur in repeat elements. A similar single large deletion has been reported in the MCPH1 microcephalin gene^32^ and a translocation dissecting *ASPM* has previously been reported.^33^ The mutations were spread throughout the gene with no hot-spots (fig 1). There were no clear cut genotype/phenotype correlations of head size centile, degree of mental retardation (profound mental retardation was not seen, nor has it been reported in *ASPM* MCPH) or the presence of epileptic fits with mutation type or position within the gene.^6^ However, only a few individuals in these cohorts have had an ethically appropriate IQ test. The findings that the mutations were spread throughout the *ASPM* gene, that all but one of the mutations led to premature protein truncation, and that there are no genotype/phenotype correlations, argue that the disease mechanism causing MCPH is nonsense mediated decay of the *ASPM* mRNA leading to a pronounced reduction of the protein in neuroepithelial cells.^16^ In support of this, two studies have looked at non-neuroepithelial cells from *ASPM* MCPH patients and found a combination of absent or much reduced levels of ASPM protein and truncated versions of ASPM still containing the C-terminus.^14^ Presumably these truncated forms of ASPM arise from atypical splicing events and/or translation re-initiation. If these results can be extrapolated to neuroepithelial cells undergoing neurogenic mitosis, then this argues that the MCPH phenotype only occurs when ASPM falls below a critical level.

The role of missense mutations in *ASPM* remains obscure. We found numerous sequence variants in study families whether they had a premature protein truncating mutation or not (supplemental table 3). Some of these were known single nucleotide polymorphisms (SNPs), some we found recurrently and some were unique to one family, but none would be predicted to cause a loss of function. For the majority of families we could show that parents were heterozygous for these sequence variants, at least ruling out a dominant negative effect (and the region is not known to be imprinted). An *ASPM* missense mutation has been previously reported,^34^ and while it was the only mutation found in an MCPH5 linked family it occurred in one of the 70+ IQ domains of the ASPM protein, and its functional significance has not been investigated. So at present there is no evidence that missense *ASPM* mutations cause MCPH; however, it remains a possibility that missense mutations may cause another phenotype or could cause autosomal dominant microcephaly.

The first aim of this study was to re-examine the current MCPH clinical criteria. We thought this necessary as the initial MCPH criteria were defined in a research setting and we wanted to see if these criteria were sufficiently robust to allow early and accurate diagnosis. We achieved this by examining which cases had *ASPM* mutations. Cohort 1 and 2 were ascertained using the current criteria and in both a similar high rate of *ASPM* mutations was found (39% and 40%, respectively). This is particularly important as the two cohorts have different ethnic and geographical origins as well as cohort 1 being inbred in contrast to cohort 2, which was not. So in these two diverse groups the current criteria was an excellent discriminator for *ASPM* mutations. If these data are taken with previous segregation and clinical analyses which suggest that three quarters of carefully selected cases of primary microcephaly have MCPH, we can conclude that *ASPM* is the most common cause of MCPH and accounts for one half of Asian and European Caucasian MCPH cases. In contrast, when diagnostic criteria were relaxed (as in cohort 3) to microcephalic children with mental retardation, but not fulfilling the other MCPH criteria, significantly fewer mutations were found (7%, p<0.01 Student t test, 2 df). Retrospective phenotype analysis showed ASPM mutations were only found in individuals meeting MCPH criteria (3/13). In those who did not meet the criteria (n = 32) no ASPM mutations were found.

**Web resources**

OMIM On-line Mendelian Inheritance in Man:

http://www.ncbi.nlm.nih.gov/Omim/

We suggest one modification to add to the MCPH criteria, that while epileptic fits are not common they should not be an excluding factor. In cohort 1 there were tonic–clonic fits in some affected individuals in two families with *ASPM* mutations; two out of three affected in one family and both children in a second family. The onset was after the first year of life, status epilepticus did not occur, there was no regression associated with the fits and they were easily controlled with standard medications. A family with primary microcephaly and epileptic fits and a protein truncating *ASPM* mutation has been previously reported.^35^ The *ASPM* mutations that have been associated with epileptic fits are shown in table 1. A remaining clinical problem is the necessity for mental retardation in the diagnostic criteria of MCPH. This cannot always be reliably diagnosed in the first few years of life, although a clinician may be asked to comment on the diagnosis and prognosis of a microcephalic baby or young child. Significant microcephaly (OFC <−4 SD) alone can co-occur with normal intelligence in a number of settings—for example, Bloom syndrome (OMIM 210900), osteodysplastic primordial dwarfism due to pericentrin mutations (OMIM 210720), and autosomal dominant microcephaly (OMIM 156580).^36^ There are no reports of *ASPM* mutations associated with microcephaly and normal intelligence, and we found no examples of this in the families of this study. Therefore, positive *ASPM* testing in a microcephalic baby would be predictive of mental retardation.

The data in this study establish *ASPM* mutations as the most common cause of MCPH, and that there are no genotype/phenotype correlations. The data also indicate that *ASPM* mutations are restricted to individuals with an MCPH phenotype, and suggest *ASPM* testing in primary microcephaly is clinically of use.
